# Harnessing galactose oxidase in the development of a chemoenzymatic platform for glycoconjugate vaccine design

**DOI:** 10.1016/j.jbc.2021.101453

**Published:** 2021-11-25

**Authors:** Jeremy A. Duke, Amy V. Paschall, John Glushka, Andrew Lees, Kelley W. Moremen, Fikri Y. Avci

**Affiliations:** 1 Department of Biochemistry and Molecular Biology, University of Georgia, Athens, Georgia, USA; 2 Center for Molecular Medicine, University of Georgia, Athens, Georgia, USA; 3 Complex Carbohydrate Research Center, University of Georgia, Athens, Georgia, USA; 4 Fina Biosolutions, LLC, Rockville, Maryland, USA

**Keywords:** glycoconjugate vaccine, galactose oxidase, capsular polysaccharide, vaccine development, *Streptococcus pneumoniae*, pneumonia, CPS, capsular polysaccharide, GOase, galactose oxidase, IPD, invasive pneumococcal disease, MHCII, major histocompatibility complex class II, NaIO_4_, sodium periodate, OPA, opsonophagocytic killing potential assay, Spn, *Streptococcus pneumoniae*

## Abstract

In the preparation of commercial conjugate vaccines, capsular polysaccharides (CPSs) must undergo chemical modification to generate the reactive groups necessary for covalent attachment to a protein carrier. One of the most common approaches employed for this derivatization is sodium periodate (NaIO_4_) oxidation of vicinal diols found within CPS structures. This procedure is largely random and structurally damaging, potentially resulting in significant changes in the CPS structure and therefore its antigenicity. Additionally, periodate activation of CPS often gives rise to heterogeneous conjugate vaccine products with variable efficacy. Here, we explore the use of an alternative agent, galactose oxidase (GOase) isolated from *Fusarium* sp. in a chemoenzymatic approach to generate a conjugate vaccine against *Streptococcus pneumoniae*. Using a colorimetric assay and NMR spectroscopy, we found that GOase generated aldehyde motifs on the CPS of *S. pneumoniae* serotype 14 (Pn14p) in a site-specific and reversible fashion. Direct comparison of Pn14p derivatized by either GOase or NaIO_4_ illustrates the functionally deleterious role chemical oxidation can have on CPS structures. Immunization with the conjugate synthesized using GOase provided a markedly improved humoral response over the traditional periodate-oxidized group. Further, functional protection was validated *in vitro* by measure of opsonophagocytic killing and *in vivo* through a lethality challenge in mice. Overall, this work introduces a strategy for glycoconjugate development that overcomes limitations previously known to play a role in the current approach of vaccine design.

While glycoconjugate vaccines have provided great health benefits in controlling bacterial diseases, their chemical conjugations have often been empirical, with weakly controlled conjugation chemistries resulting in poorly characterized, heterogeneous, and variably immunogenic glycoconjugates. Especially problematic has been the inadequate understanding of the mechanisms by which the immune system responds to these complex vaccines. *Streptococcus pneumoniae* (*Spn*) is a human pathogen for which glycoconjugate vaccine immunization has been utilized clinically. This Gram-positive bacterium acts as the main causative agent for invasive pneumococcal diseases (IPDs) worldwide and maintains its status as a major health risk to both pediatric and elderly populations despite advances in vaccine development spanning decades. Current pneumococcal vaccine formulations are designed to provide protection against individual serotypes of *Spn* by eliciting an immune response specific to the capsular polysaccharide (CPS) coating the surface of the bacterium. The distinct structural composition of the CPS serves as a unique identifier for individual pneumococcal serotypes and functionally serves the bacteria as the primary virulence factor that allows for colonization as well as immune evasion and resistance within the host (1, 2). Generation of a long-lasting and specific immune response to the CPS is difficult, as polysaccharide structures are largely T-cell independent antigens due to their inability to be presented by the Major Histocompatibility Complex class II (MHCII) molecules of antigen-presenting cells for T cell recognition (3, 4). However, through covalent linkage of CPS to a protein carrier in the formation of a glycoconjugate, the construct can be processed in the endolysosome into a molecule capable of MHCII presentation while still maintaining structural identity of the CPS for recognition by CD4+ T cells and triggering of the adaptive immune response (5, 6, 7).

Although utilization of conjugate vaccines has greatly shown a positive impact on incidence rates, multiple difficulties in their technical development have hampered overall efficacy (8, 9). One central issue lies in the synthetic methodologies employed to functionalize CPS with the necessary chemical handles or linkers needed before conjugation to a carrier can take place. One of the most common strategies involves treatment of CPS with NaIO_4_ to act on the vicinal diols within the polysaccharide structure, generating reactive aldehydes (10, 11). However, this experiential approach is a double-edged sword, as the reaction will be performed in a poorly controlled fashion potentially opening many sugar rings along the CPS chain irreversibly. This will not only result in a polysaccharide susceptible to further degradation but also will likely generate many “scars” on the polysaccharide structure preventing antibody cross-reactivity with the native polysaccharide on the surface of the infecting bacterium (12). The reported use of large ranges of oxidant highlights how variable conjugate formulations will be, as seen by the large degree of batch variability and immunological evaluation differences known to play a factor in conjugate vaccine manufacturing (13). To combat this poorly controlled approach of activating the CPS, we have employed the galactose oxidase enzyme isolated from *Fusarium* sp. as an enzymatic platform to introduce aldehyde motifs in the CPS in a manner that is both site-specific and reversible. GOase is a widely studied oxidase capable of acting on monosaccharide and terminal-end galactose residues to oxidize the C6 primary hydroxyl into an aldehyde (14, 15). Since GOase has the unique ability to selectively oxidize galactose residues, we explored whether pneumococcal CPS containing branching galactose within its structure would act as a substrate for oxidation, functionalizing the CPS for chemical conjugation. Here, we show that GOase can oxidize bacterial CPS using Pn14p as a representative pneumococcal CPS. This holds significance as Pn14p is included in currently licensed glycoconjugate vaccines and is traditionally activated solely through chemical oxidation (16). We further investigate the capacity of harnessing this approach in the formation of glycoconjugates, ultimately evaluating the immunological efficacy of the conjugate vaccine developed through *in vitro* correlates of protection and live, virulent type 14 *S. pneumoniae* lethality challenge *in vivo*.

## Results

### Chemoenzymatic derivatization of Pn14p

**Figure 1:**
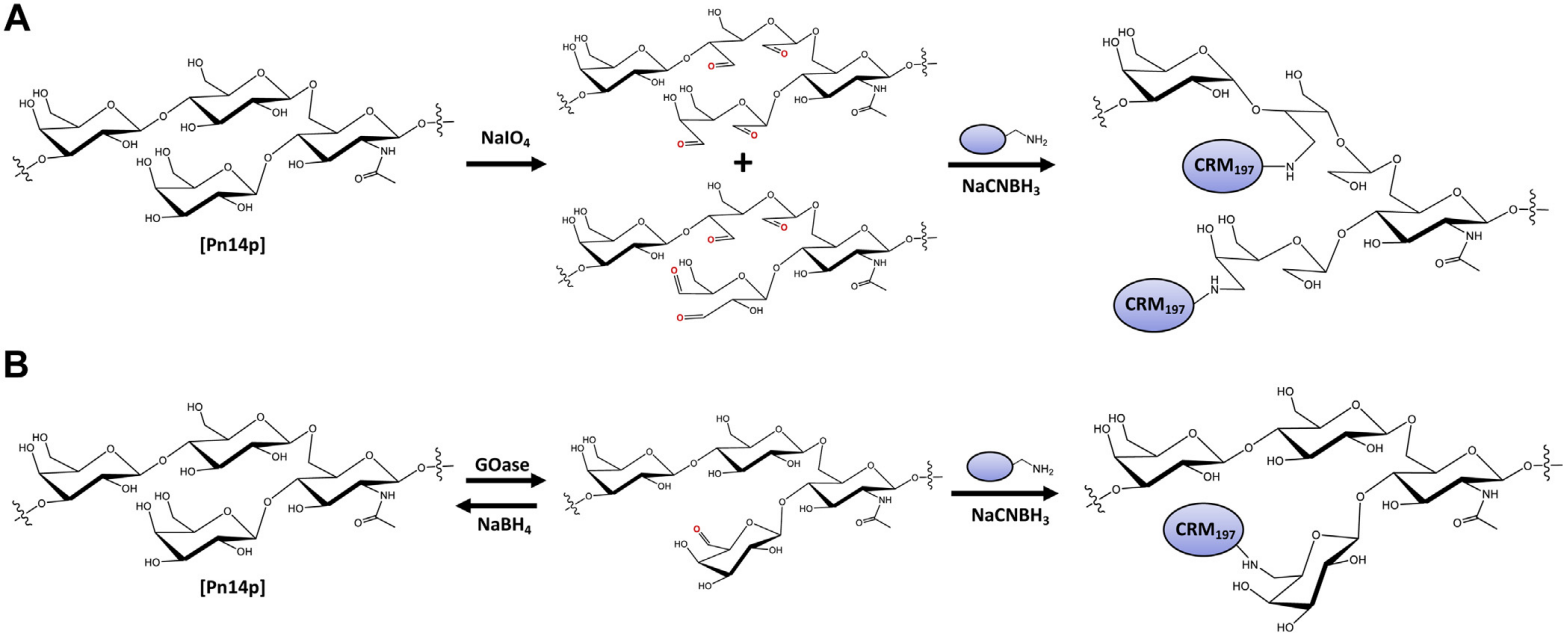
**Highlighting the structural differences between the chemical and chemoenzymatic conjugation strategies.***A*, in the traditional chemical approach harnessing sodium periodate, poorly controlled activation along the CPS structure results in the permanent opening of the vicinal diols prior to conjugation to carrier. The varying formation of aldehydes results in random conjugations with the carrier protein further contributing to structural heterogeneity of the glycoconjugate vaccine. *B*, harnessing GOase as an enzymatic approach selectively modifies the C6 hydroxyl on branching galactose residues in a manner that does not change the core structure prior to conjugation. After reductive amination, any nonconjugated aldehydes would be converted back to the hydroxyl with sodium borohydride treatment maintaining native structural identity. CPS, capsular polysaccharide; GOase, galactose oxidase.

To assess the difference between a traditional chemical strategy harnessing NaIO_4_ oxidation to derivatize CPS as compared with the enzymatically directed oxidation through galactose oxidase, a representative pneumococcal polysaccharide found in the current pneumococcal conjugate vaccine (Prevnar 20; Pfizer, Inc) was selected. *Spn* serotype 14 synthesizes the CPS with a tetrasaccharide repeating unit containing a terminal galactose branching from the N-acetylglucosamine on the trisaccharide backbone [β-D-Galp(1→4)]→ 6)-β-D-GlcpNAc-(1→ 3)-β-D-Galp-(1→ 4)-β-D-Glcp-(1→ (Fig. 1) (17). This terminal galactose residue can be selectively targeted and reversibly oxidized on the C6 hydroxyl position using galactose oxidase followed by treatment with a reducing agent, NaBH_4_. In contrast, periodate oxidation of Pn14p results in poorly selective chemical oxidation of vicinal diols as is necessary using periodate treatment (Fig. 1). We first expressed the GOase enzyme in *E. coli* as previously described and demonstrated the ability to use Pn14p as a substrate foroxidation with purified GOase as determined through a colorimetric assay measuring oxidase activity (18, 19, 20) (Fig. S1).

### Characterization of the reversible GOase oxidation of Pn14p

**Figure 2:**
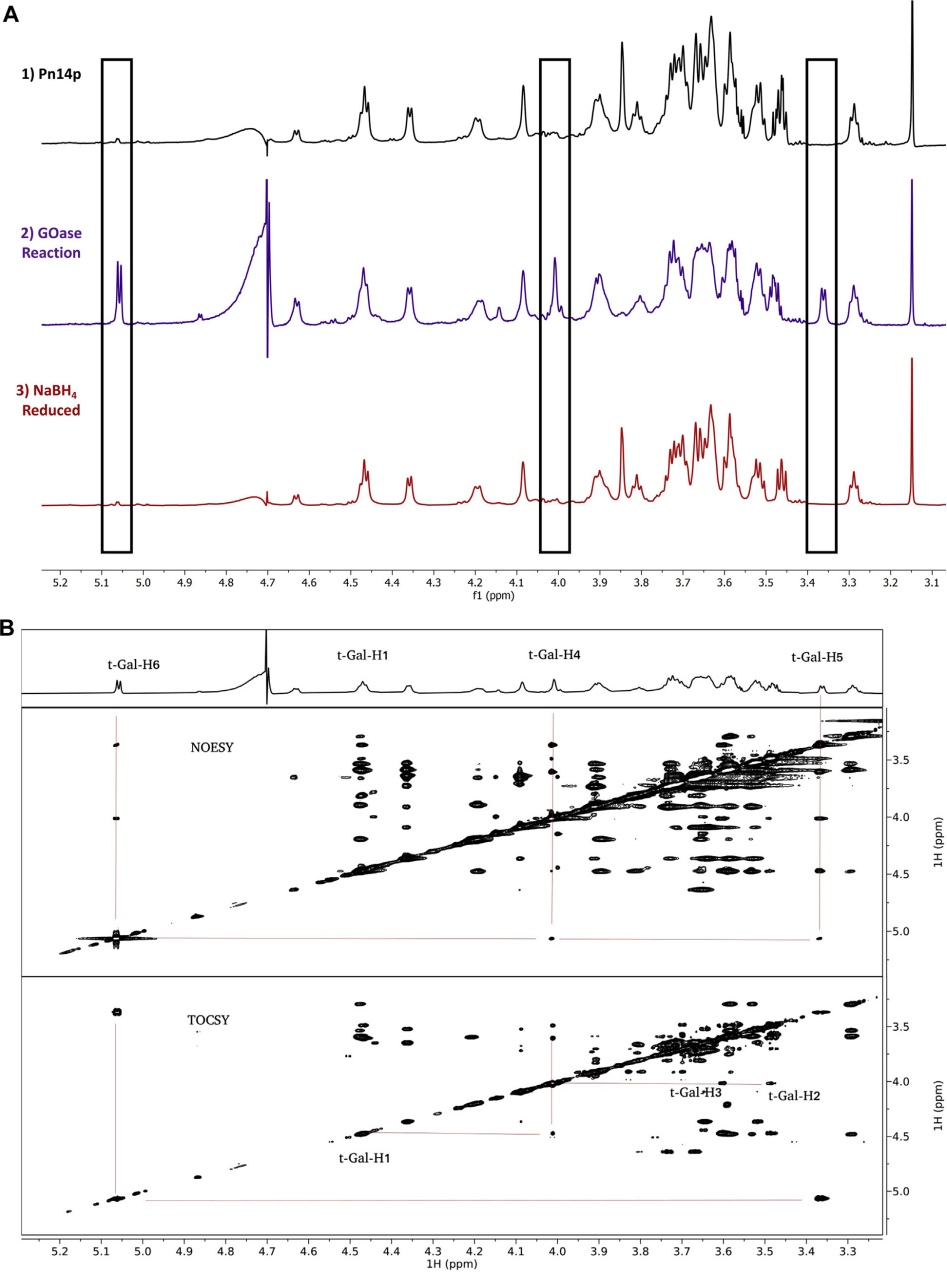
**NMR characterization of reversible GOase activation of Pn14p.***A*, 1D ^1^H spectra of three samples corresponding to Pn14p, GOase oxidized Pn14p, and GOase oxidized Pn14p that has been treated with NaBH_4_. *B*, 2D TOCSY and NOESY spectra of the GOase oxidized Pn14p highlighting the 6-oxogalacto configuration. GOase, galactose oxidase.

To verify the selective oxidation of Pn14p by GOase as well as its reversibility to the native form of the polysaccharide, we performed nuclear magnetic resonance (NMR) spectroscopy experiments. The NMR samples consisted of (1) unmodified, purified Pn14p dissolved in sodium bicarbonate D_2_O buffer, (2) Pn14p in the same buffer after reaction with galactose oxidase, and (3) Pn14p reacted with galactose oxidase as in (2), then reduced with NaBH_4_ before dialyzing into D_2_O. The middle trace of Figure 2*A* shows the presence of three clearly distinct resonances (H6 hydrate, 5.06 ppm; H4, 4.01 ppm; H5, 3.36 ppm) in the oxidized sample, fitting the expected assignments of terminal 6-oxogalactose within Pn14p (14, 21). These signals were further confirmed to be consistent with the hydrated form of 6-oxogalactose structure by analysis of the 2D TOCSY and NOESY spectra (Fig. 2*B*). The bottom TOCSY panel shows connectivity between H6 and H5, then connectivity between H1 through H4, typical of galacto configuration. The middle NOESY panel confirms the galacto configuration with NOE cross peaks between H5, H6, and H4. The removal of these signals is confirmed in the third trace of Figure 2*A*, postreduction by NaBH_4_. These results indicate the successful formation of a site-selective aldehyde on Pn14p, with conversion back into the native form. This verifies the ability for the chemoenzymatic method to activate Pn14p without the tentatively deleterious effects in the haphazard activation of polysaccharide through chemical oxidation.

### Enzymatic derivatization of Pn14p is significantly less destructive than chemical oxidation

To assess the functional outcomes of GOase activation of Pn14p, we directly compared GOase-derivatized Pn14p with chemical derivatization with NaIO_4_ using two different concentrations of oxidant, either 0.25 or 1 mol equivalent (mol.Eqv.) of oxidant per repeat unit contained within Pn14p. While the oxidation conditions used in the commercial Pn14p conjugate are not clearly defined, based on previous literature and our own experience, 1 mol equivalent use of NaIO_4_ results in a sufficiently oxidized polysaccharide to yield a conjugation product with roughly equal weight content of protein and polysaccharide (16, 22, 23, 24).

**Figure 3:**
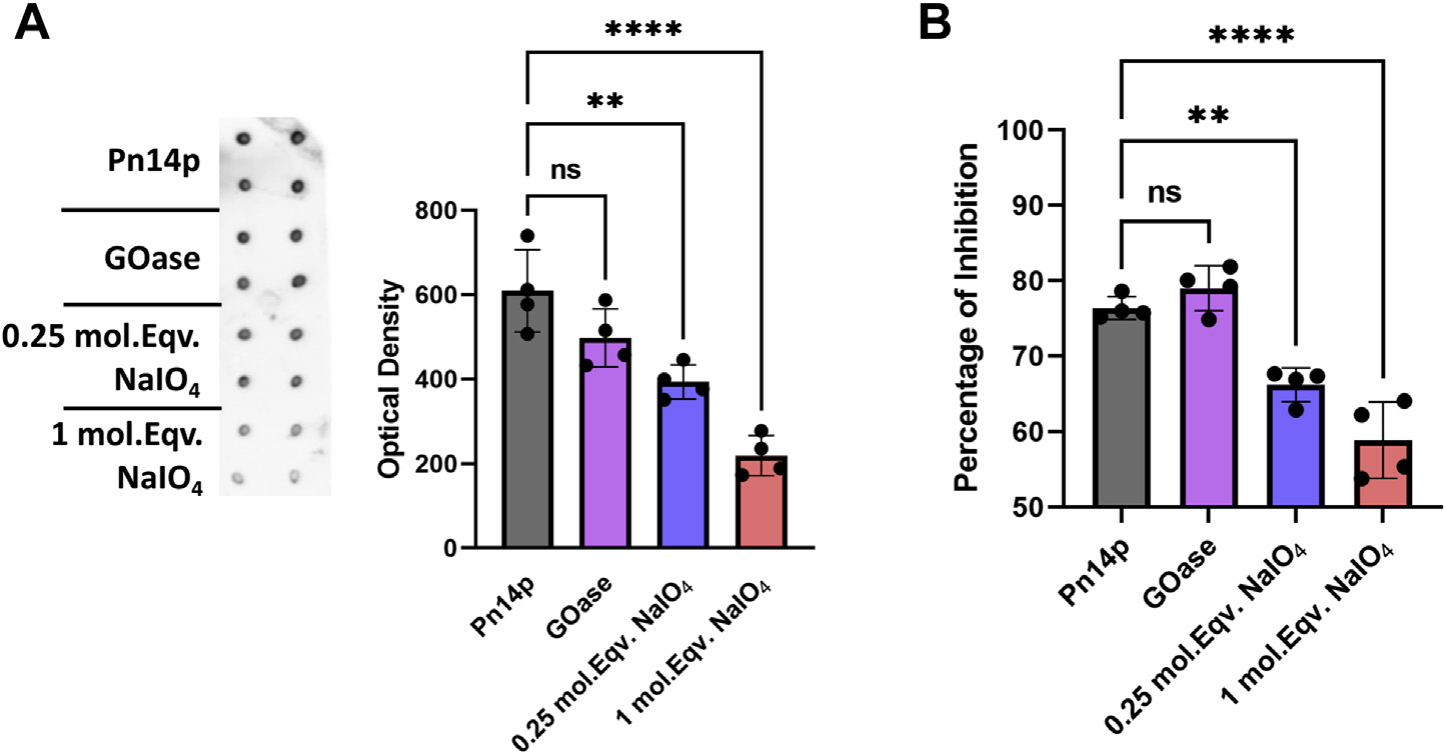
**Functional comparison between GOase and periodate oxidation of Pn14p.** Pn14p was treated with either GOase or one of two different concentrations of sodium periodate, relating to 0.25 or 1 mol equivalent of NaIO_4_ to 1 mol of CPS repeat unit. *A*, a dot blotting assay was performed on the polysaccharides in quadruplicate using Anti-Pn14p IgG with the optical density of the samples measured using the NIH ImageJ software. *B*, the polysaccharides were then used in a competition ELISA in which samples were incubated in quadruplicate with the anti-Pn14p polyclonal IgG prior to introduction to a Pn14p coated plate. Values represent the mean ± SD of the optical density or percentage of inhibition, respectively. ∗∗∗∗*p* < 0.0001 and ∗∗*p* < 0.01 represent significant differences between groups determined using one-way ANOVA with Dunnett's multiple comparisons test. CPS, capsular polysaccharide; GOase, galactose oxidase.

Through dot blotting using polyclonal serum from mice immunized with a type 14 conjugate made with CDAP cyanylation chemistry as a third, orthogonal method (25), we assessed the antibody recognition of the GOase-treated polysaccharide in comparison to native Pn14p. While GOase-treated Pn14p similarly reacted with the Pn14p polyclonal serum as with purified, unmodified Pn14p, NaIO_4_-treated Pn14p showed significantly reduced reactivity (Fig. 3*A*). This finding was further validated using a competition ELISA with Pn14p-coated plates, resulting in no significant difference between unmodified Pn14p and GOase-treated Pn14p in its ability to inhibit antibody binding compared with reduced competitive inhibition using NaIO_4_ derivatized CPS (Fig. 3*B*). These results indicate that GOase treatment results in selective activation of the polysaccharide with minimal decrease in the structural integrity of the CPS and thus highly akin to the native form of the type 14 CPS that the immune system would encounter in infection.

### Production of a chemoenzymatic pneumococcal glycoconjugate vaccine

Having observed the efficient and selective activation of Pn14p using GOase, we sought to determine if this approach would yield an immunogenic glycoconjugate vaccine comparable to one generated using periodate oxidation. To this end, Pn14p was first activated with GOase and purified using Size Exclusion Chromatography (SEC) followed by conjugation to CRM_197_ through reductive amination. The conjugation product was then purified using SEC, with a marked shift in the absorbance at 280 nm indicative of the formation of a high-molecular-mass glycoconjugate, as compared with a sample containing unconjugated Pn14p mixed with CRM_197_ (Fig. S2*A*). An additional glycoconjugate was prepared for comparison using sodium periodate (1 mol equivalent per polysaccharide repeating unit) oxidation following the purification described above. The formation of glycoconjugate was validated through dot blotting using polyclonal serum from mice immunized with CRM_197_ to confirm the collected high-molecular-weight fractions contained CPS conjugated carrier protein and were recognized by the anticarrier antibodies, whereas polysaccharide alone did not react (Fig. S2*B*). These conjugates were then analyzed for their protein and polysaccharide content using the bicinchoninic acid (BCA) and phenol–sulfuric acid assays, respectively (Fig. S2*C*). From these results it was found both conjugates have a comparable level of protein and polysaccharide weight contents. Conjugation with 0.25 mol equivalent NaIO_4_ activation of the polysaccharide yielded a very poorly conjugated product (<5% protein content using BCA).

### Humoral response generated by a chemoenzymatically synthesized glycoconjugate

**Figure 4:**
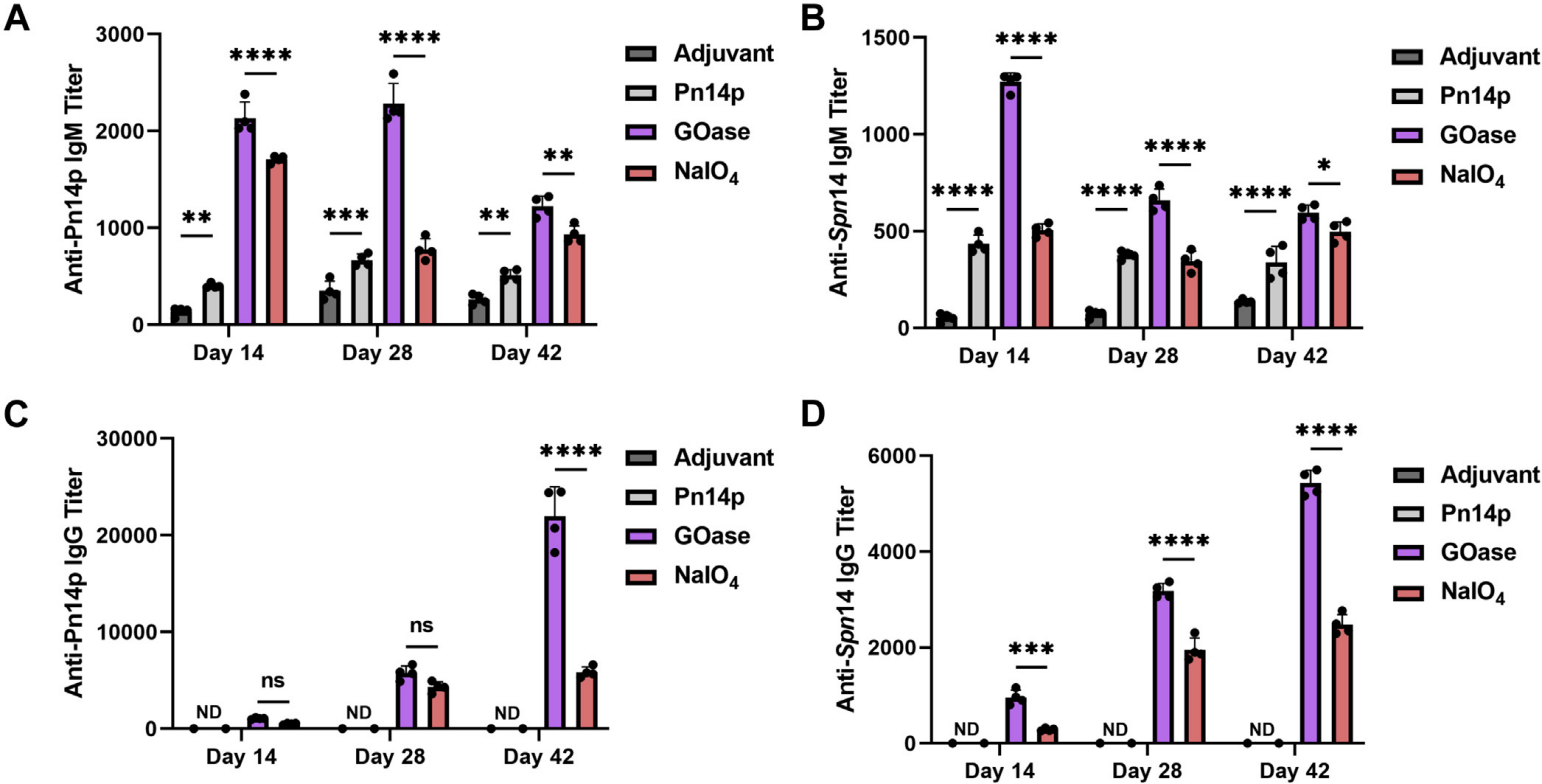
**The effect activation strategy has on IgM and IgG antibodies generated.** Mice (n = 5) were primed (Day 0) and boosted on day 14 and 28, with sera collected 2 weeks after each vaccine administration to calculate kinetics of the humoral response generated with each sample tested in quadruplicate. Pn14p (*A* and *C*) and fixed whole-bacteria *S. pneumoniae* expressing the type 14 capsule (*B* and *D*) specific antibody responses from each time point were measured using ELISA. Regression curves based on individual serum dilutions were used to determine IgG and IgM titer levels at an OD of 0.5. Shown are calculated mouse titers as the reciprocal dilution at OD of 0.5 at 405 nm in the ELISA assay. Values represent the mean ± SD of the IgM or IgG titers, respectively. ∗∗∗∗*p* < 0.0001, ∗∗∗*p* < 0.001, and ∗*p* < 0.05 represent significant differences between groups determined using two-way ANOVA with Dunnett's multiple comparisons test.

The GOase and NaIO_4_ derivatized conjugates were then tested for their capability to induce a humoral immune response. Groups containing five BALB/c mice each were vaccinated using a preparation of conjugate with Alum adjuvant at a dosage corresponding to 2 μg CPS content per mouse. Two additional control groups consisting of adjuvant alone and Pn14p with adjuvant were included in the study. Mice were immunized three times intraperitoneally (i.p.) at 2-week intervals, with sera collected and analyzed as individual mouse sera for serotype-specific IgM and IgG antibody responses at days 14, 28, and 42. Characterization of the antibody responses using the murine sera was performed by ELISA, using plates that had either been coated with purified Pn14p or fixed whole-bacteria of a serotype 14 specific strain of *S. pneumoniae* ((Klein) Chester 6314). In both purified CPS- and whole bacteria-coated ELISAs, IgM levels against CPS were significantly higher in the conjugate vaccine groups on day 14, after the primary immunization (Fig. 4, *A* and *B*). GOase conjugate elicited a statistically significant higher level of IgM as compared with the NaIO_4_ group on each day tested. The kinetics of an increasing IgG response over time was seen in both conjugate vaccine groups, whereas no detectable IgG antibodies were observed in either control group (Fig. 4, *C* and *D*). Notably, the GOase conjugate elicited a robust level of IgG at the day 42 timepoint compared with the NaIO_4_ group, with an almost fourfold higher antibody titer when using plates coated with Pn14p and twofold higher when using fixed serotype 14 bacteria. Together, these results highlight the immunogenic importance the method of CPS activation has in terms of humoral response. As part of these immunizations, we also used the conjugate prepared with 0.25 mol equivalent sodium periodate, but due to its very low protein incorporation, it showed a similar antibody response as the polysaccharide alone group (not shown).

### Vaccine-induced functional protection activity

**Figure 5:**
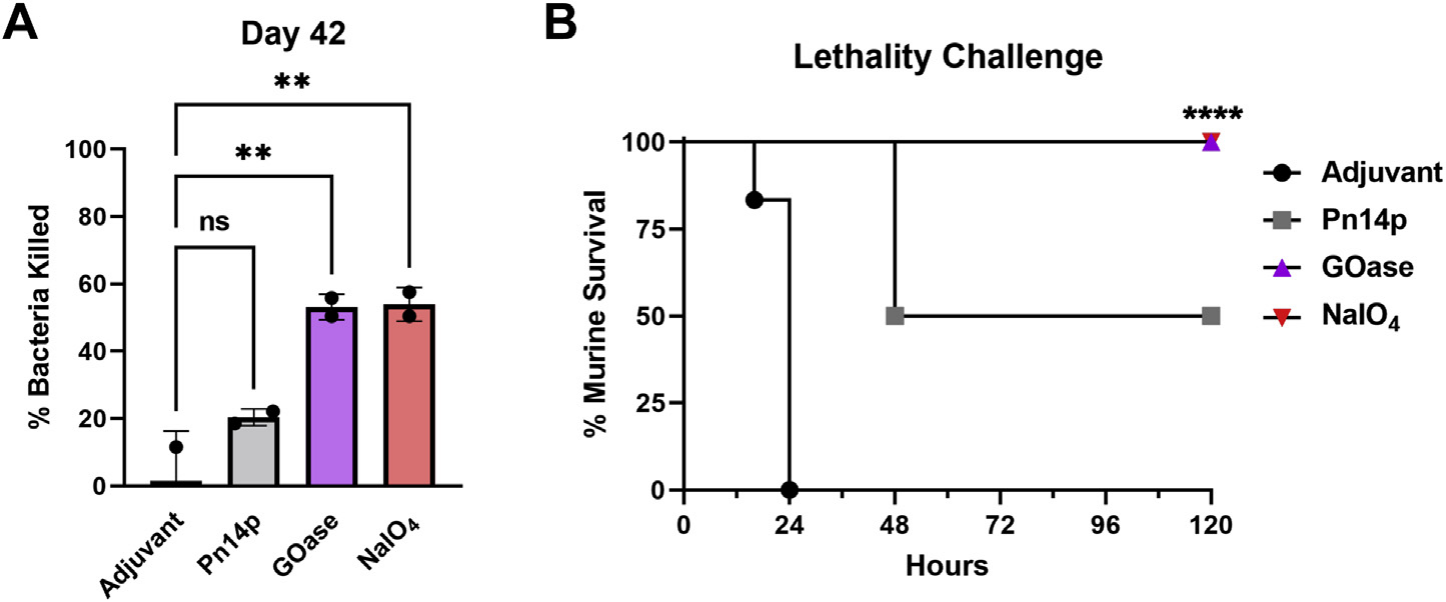
**Functional protection elicited through glycoconjugate vaccination.***A*, using serum from each sample group at day 42, opsonic capacity of antibodies was determined against live *Spn*14, with the correlated degree of bacteria killed from an OPA shown. *B*, survival of vaccinated mice (n = 6) challenged on day 49, 3 weeks after the final boost, by virulent *Spn*14. Values (*A*) represent the mean percent ±SD of the bacteria killed or (*B*) murine survival. ∗∗∗∗*p* < 0.0001 and ∗∗*p* < 0.01 represent significant differences between groups determined using one-way ANOVA with Dunnett's multiple comparisons test or survival curve comparison using Mantel–Cox Log-rank test.

To evaluate protection elicited against live encapsulated *Spn* after introduction of the experimental vaccine, we first tested for functional antibodies *in vitro* using an opsonophagocytic killing potential assay (OPA) with the endpoint sera collected on day 42 from each of the sample groups (Fig. 5*A*). From these results it was seen that a comparable degree of bactericidal activity occurred when using either the chemical or chemoenzymatically generated conjugate vaccine, with both glycoconjugates killing a statistically significant amount (>50%) of serotype 14 *Spn* compared with adjuvant injection control. In a separate experiment, sample groups were also tested for *in vivo* protection using a murine sepsis infection model against the same serotype 14 *Spn* strain 3 weeks after the final boost, on day 49. To each mouse, a relatively high (∼1.5 × 10^8^ CFU; ∼6× LD_50_) (26) dose of *Spn*14 was given i.p. for a lethality challenge. All adjuvant immunized mice were moribund within 24 h after administration and euthanized (Fig. 5*B*). In contrast, all conjugate vaccine administered mice showed minimal symptoms relative to adjuvant mice, with a 100% survival rate over the course of study. In both functional protection studies, the Pn14p group did show activity, which can be attributed to the IgM response elicited through immunization with CPS alone—an effect that is used clinically for short-term protection against encapsulated pneumococcus currently (27). Taken together, these results show that a novel chemoenzymatic approach for reversible activation of CPS can be harnessed to form glycoconjugate vaccines that are site-specific and nondestructive for the competent polysaccharide antigen and is capable of providing a more robust humoral response with equal protection as the type found in commercial preparations.

## Discussion

Glycoconjugate vaccines have had a profound benefit to human health through the prevention of encapsulated bacterial infection since their introduction decades ago, but the manufacturing and production have been knowingly met with complications resultant from the often empirical and ill-defined approaches used in the development of conjugate molecules (5, 8, 12). One issue that is known to play a role in the variability of glycoconjugate efficacy is the synthetic methodologies used to derivatize CPS structures for conjugation to a carrier protein due to the lack of a ready chemical handle on most bacterial polysaccharides. The most employed approach for CPS derivatization is the formation of aldehyde groups that can then be conjugated directly to lysine residues on carrier proteins or modified with a linker molecule (28). To generate these aldehyde motifs, sodium periodate is primarily used to chemically oxidize vicinal diols found throughout the CPS structures. It is well established that efficiency of periodate oxidation is dictated by the nature of the diol structures found within the polysaccharide (29, 30). The oxidant strongly prefers acyclic vicinal diols, such as those found in group B *Streptococcus* serotype III sialic acids to oxidize quantitatively (28), whereas excess amount of oxidant as well as longer reaction times and higher temperatures may be needed to successfully oxidize cyclic diols (12, 22). Most CPS structures, including Pn14p, contain cyclic vicinal diols that would not readily react with sodium periodate, necessitating a larger amount of oxidant be used to generate a sufficient degree of saccharide activation using the diols found within the rings of the sugars that compose the capsule. The excess periodate concentration coupled with poorly controlled oxidation reaction may result in significant structural changes and the degradation of CPS (11, 12). Recent research into the parameters of conjugate vaccine design that help drive high efficacy has shown that consistent presentation of CPS epitopes is essential in generation of a robust immune response, highlighting the need for more specific and nondestructive approaches in the synthetic strategies employed by conjugate vaccine manufacturers. We investigated a chemoenzymatic method to modify CPS structure in a site-selective and reversible fashion that alleviates concerns of harnessing tentatively deleterious chemical approaches that largely act randomly, using serotype 14 of *Spn* as a proof-of-principle model system. We further show that this approach can induce a significantly greater humoral immune response compared with a traditional chemical conjugate, with *in vitro* and *in vivo* functional assays validating protective immunity against pneumococcal infection.

GOase has been used for multiple applications, from biological sensors to organic synthesis (15, 31, 32, 33). In this study we postulated an effective and important use that would have widespread application in the field of conjugate vaccine manufacturing by enzymatic modification of CPS structures, without being limited to a singular serotype of encapsulated bacteria. For the purposes of this study, serotype 14 was chosen for its prevalence in invasive pneumococcal infection despite inclusion in both the 13-valent conjugate and 23-valent polysaccharide vaccines commercially available, known emergence of antibiotic resistance strains, and accessibility of galactose in the CPS (1, 34, 35, 36, 37). Of the 24 *Spn* serotypes included in current commercially available pneumococcal vaccines, GOase would be able to readily activate 9, or roughly 37.5%, of CPS structures as determined by the presence of a terminal galactose residue within the repeat unit of the CPS (38, 39), with current ongoing work assessing engineered variants of GOase to further expand the repertoire of structures to nongalactose-containing substrates. One such direct evolution product, GOase_F2_, has been previously reported to act on a range of terminal hexoses (18). Based on CPS structure, this mutant enzyme can potentially activate another seven of the 24 CPSs included in the available pneumococcal vaccines and 19 nonincluded serotypes (38, 39). Moreover, polysaccharide antigens from a vast variety of human pathogens such as *Neisseria meningitidis*, *Streptococcus flexneri*, and *Streptococcus agalactiae* are potential targets using this chemoenzymatic approach (40, 41, 42). A key difference in our chemoenzymatic approach compared with previous chemical methods lies in its ability to be both site-selective and reversible, without dependence on deleterious oxidation strategies that will result in structural changes. In our approach, all nonconjugated sites revert to their antigenic form as native Pn14p (Fig. 2). We believe this reversibility of oxidation is a major strength of GOase over periodate. In addition, unlike periodate oxidation, GOase oxidation does not result in ring-opening that renders the CPS susceptible to degradation.

Here, we developed a chemoenzymatic conjugation platform to design and produce a prototype conjugate vaccine that is structurally better defined and superior in function compared with its traditionally prepared counterpart. Our results illustrate an approach in the development of pneumococcal vaccines that has broader impact in the field of glycoconjugate vaccines, with many encapsulated pathogens expressing a CPS that can be targeted for modification by GOase or an engineered mutant. Knowledge-based vaccine design may pave the road for a new generation of conjugate vaccines that are structurally and functionally well defined with high clinical efficacy.

## Experimental procedures

### Antigens

*E. coli* expressed CRM_197_ (EcoCRM) was provided by Fina Biosolutions and used as the carrier protein in the formation of the antigens. Purified Pneumococcal Type 14 capsular polysaccharide (Pn14p) was purchased from ATCC. Before use, polysaccharide was brought to an average molecular mass of ∼300 kDa through mild ozonolysis using a portable ozone generator, by first dissolving the polysaccharide in sodium bicarbonate buffer before treating with ozone for 3 min at a rate of 10 mg per minute. The polysaccharide was then purified by size exclusion chromatography column with detection by refractive index (ENrich SEC 650 10 × 300 Column) with size determined relative to dextran polysaccharide standards.

*S. pneumoniae* type 14 (Klein) Chester 6314 was purchased from ATCC and grown on tryptic soy agar plates supplemented with 5% sheep blood overnight at 37 °C. Colonies were plucked and grown in Todd-Hewitt broth with 2% yeast extract at 37 °C.

### Galactose oxidase expression and purification

The gene for expression of *Fusarium* sp. derived Galactose Oxidase was subcloned into a pET vector backbone as a sfGFP-6xHis fusion protein. BL21 competent *E. coli* from New England BioLabs was transformed using the plasmid with ampicillin resistance inserted for colony selection. Colonies were grown in LB broth at 190 RPM, 37 °C until an optic density of 0.6 at 650 nm was observed. The bacterial culture was then moved to 18 °C and supplemented with 100 mM of IPTG to begin expression. After 16 h, the bacteria were collected by centrifugation, washed, and lysed, with purification of GOase performed through standard IMAC protocol.

### Enzymatic activity assay

Galactose oxidase activity was measured as previously described, using an indirect peroxidase-coupled assay to provide a colorimetric readout based on H_2_O_2_ generated during the reaction (18). Briefly, 10 μg of GOase enzyme was added to a reaction vessel containing 1 U/ml horseradish peroxidase, 100 μg ABTS, and 10 μg substrate in 100 mM phosphate buffer (pH 7.4). Absorbance was measured periodically at 420 nm. Experiments were performed in quadruplicate.

### Conjugate vaccine production

Size fractionated Pn14p was dissolved in deionized water, to which 1 M equivalent amount of sodium metaperiodate per polysaccharide repeating unit was added and the reaction allowed to react at 30 °C for 1 h. The periodate oxidation was then quenched by adding molar excess of ethylene glycol to use any remaining periodate, before dialyzing the product against deionized water for 24 h. After dialysis, the periodate oxidized Pn14p was lyophilized. After lyophilization, 1 mg of oxidized polysaccharide and EcoCRM was dissolved in 700 μl sodium bicarbonate buffer, to which 10 mg of sodium cyanoborohydride was added and the pH adjusted to 8. The reaction was performed for 48 h at 37 °C, after which the reaction was dialyzed against deionized water and lyophilized. The product was purified and characterized using an ENrich SEC 650 10 × 300 column by a shift in the absorbance at 280 nm and refractive index, indicating the formation of a high-molecular-mass product.

In the formation of the GOase oxidized conjugate, 1 mg of size fractionated type 14 polysaccharide was dissolved in 700 μl sodium bicarbonate buffer, to which 20 μg of GOase and 1 μg of HRP were added. The reaction vessel was purged with purified oxygen to catalyze GOase activity. The vessel was kept at 37 °C and allowed to react for 8 h before being stopped by heat shocking at 99 °C for 10 min. The oxidized Pn14p was purified using size exclusion chromatography and detected by refractive index as described above and concentrated. To this concentrated polysaccharide sample, 1 mg of EcoCRM was added and volume adjusted to 700 μl followed by the addition of 10 mg of sodium cyanoborohydride. The pH of the reaction was adjusted to 8 and allowed to react for 48 h at 37 °C. Following this incubation, unreacted aldehydes were reduced back to their native alcohol by adding 2 mg of sodium borohydride and allowing the reaction to continue for 6 h at 37 °C. The reaction was then dialyzed against deionized water for 24 h and lyophilized, with conjugate formation characterized as previously described (6, 28). Conjugates were then assayed for protein and carbohydrate content using the BCA and phenol–sulfuric acid assays (43, 44).

### Nuclear magnetic resonance

NMR analyses were performed at the Complex Carbohydrate Research Center’s NMR Facility. Data were collected at 25 °C on a Bruker Neo 900 MHz spectrometer equipped with a 5 mm TXO cryoprobe. 1D proton spectra were collected using the presaturation sequence zgpr from the standard Bruker library, and the 2D NOESY and TOCSY spectra were acquired with modified noesygpphfpr and dipsi2gpphpr sequences, respectively, to allow for off center presaturation of the water signal. The NOESY mixing time was 120 ms and the TOCSY mixing time was 80 ms. Data were processed with MestReNova (Mestrelab Research). Chemical shifts were approximated by referencing to residual water at 4.7 ppm.

### Dot blot assay

Samples of Pn14p were blotted on a PVDF membrane. The membrane was incubated in 3% BSA in PBST buffer (0.1% Tween 20 in 1× PBS, pH 7.4) for 1 h followed by a brief wash in PBST. The membrane was then incubated with polyclonal Anti-Pn14p sera (1:5000 dilution in 1% BSA PBST buffer) for 2 h at room temperature followed by four 10-min PBST washes. The membrane was then incubated with horseradish peroxidase linked anti-mouse IgG secondary antibody (1:10,000 in 1% BSA PBST) for 1 h at room temperature followed by five 10-min PBST washes. The membrane was developed using chemiluminescent substrate (Bio-Rad) and imaged on an Analytik Jena UVP ChemStudio imager. Signal densitometry was performed using the NIH ImageJ software.

### Mouse immunizations

Female BALB/c mice of 6 to 8 weeks of age were immunized intraperitoneally with 100 μl of an emulsion containing 1% Alum as an adjuvant along with either 4 μg of conjugate (∼2 μg polysaccharide content), 2 μg of polysaccharide, or PBS. Mice were immunized three times, 14 days apart. All mouse experiments were in compliance with the University of Georgia Institutional Animal Care and Use Committee under an approved animal use protocol. Our animal use protocol adheres to the principles outlined in *U.S. Government Principles for the Utilization and Care of Vertebrate Animals Used in Testing, Research and Training*, the Animal Welfare Act, the *Guide for the Care and Use of Laboratory Animals*, and the *AVMA Guidelines for the Euthanasia of Animals*.

### ELISA

Mice were bled from the facial vein on days 14, 28, and 42, corresponding to 2 weeks after primary immunization, boost, and secondary boost, accordingly. Type 14 specific antibodies were determined using ELISA, with either 2 μg/ml Pn14p or 10^5^ fixed *S. pneumoniae* type 14 bacteria to coat a 384-well plate (45, 46).

### OPA

An opsonophagocytic killing assay was performed as described previously as adapted from an earlier protocol with modifications (47, 48, 49). *Spn*14 stocks were incubated in triplicate wells in a 96-well round-bottom plate for 1 h at 37 °C with the indicated sera samples (5 μl serum/50 μl total reaction volume/well) in opsonization buffer B (OBB: sterile 1× PBS with Ca2+/Mg2+, 0.1% gelatin, and 5% heat-inactivated FetalClone [HyClone]). The human promyelocytic leukemia cell line HL-60 (ATCC) was cultured in RPMI with 10% heat-inactivated FetalClone and 1% L-glutamine. HL-60 cells were differentiated using 0.6% N,N-dimethylformamide (DMF [Fisher]) for 3 days before performing the OPA assay, harvested, and resuspended in OBB. Baby rabbit complement (Pel-Freez) was added to HL-60 cells at a 1:5 final volume. The HL-60–complement mixture was added to the bacteria at 1 × 10^5^ cells/well. The final reaction mixtures were incubated at 37 °C for 1 h with shaking. The reactions were stopped by incubating the samples on ice for approximately 20 min. Then 10 μl of each reaction mixture was diluted to a final volume of 50 μl and plated onto blood agar plates in duplicate. Plates were incubated overnight at 30 °C and counted the next day. The percentage of bacterial killing was calculated as each sample replicate normalized to the mean value obtained for the control samples, subtracted from 100 (with PBS-treated control sera samples representing 0% survival).

### Lethality study

Colonies of Type 14 *S. pneumoniae* were grown in THY broth and collected at optic density of 0.3 at 650 nm, corresponding to mid-log phase. The bacterial suspension was adjusted with sterile PBS to give a count of ∼10^9^ CFU/ml as determined by viable bacterial numbers upon plating on Tryptic soy agar plates supplemented with 5% sheep’s blood. Each mouse was challenged intraperitoneally with 150 μl of the bacterial suspension, corresponding to ∼1.5 × 10^8^ CFU (∼6× LD_50_) (25). Mice were inspected every 6 h until moribundity was observed.

## Data availability

All data contained within the manuscript.

## Supporting information

This article contains supporting information.

## Conflict of interest

The authors declare no conflict of interest with the contents of this article.
