# Supplementary material for: Harnessing galactose oxidase in the development of a chemoenzymatic platform for glycoconjugate vaccine design
Source: J Biol Chem. 2021 Nov 25;298(1):101453. doi: 10.1016/j.jbc.2021.101453 (PMC8689215; doi:10.1016/j.jbc.2021.101453)
Supplement: Supplemental Figures S1 and S2 [file mmc1.docx]

**Supporting Information for**

Harnessing galactose oxidase in the development of a chemoenzymatic platform for glycoconjugate vaccine design

Jeremy A. Duke^a,b^, Amy V. Paschall^a,b^, John Glushka^c^, Andrew Lees^d^, Kelley W. Moremen^a,c^, and Fikri Y. Avci^a,b,*^

^a^Department of Biochemistry and Molecular Biology, University of Georgia, Athens, GA 30602; ^b^Center for Molecular Medicine, University of Georgia, Athens, GA 30602; ^c^Complex Carbohydrate Research Center, University of Georgia, Athens, GA 30602; and ^d^Fina Biosolutions, LLC.

* Fikri Y. Avci, 7065423831 (phone), 7065424412 (fax) **Email:** [avci@uga.edu](mailto:avci@uga.edu)

**This file includes:**

Figures S1 to S2


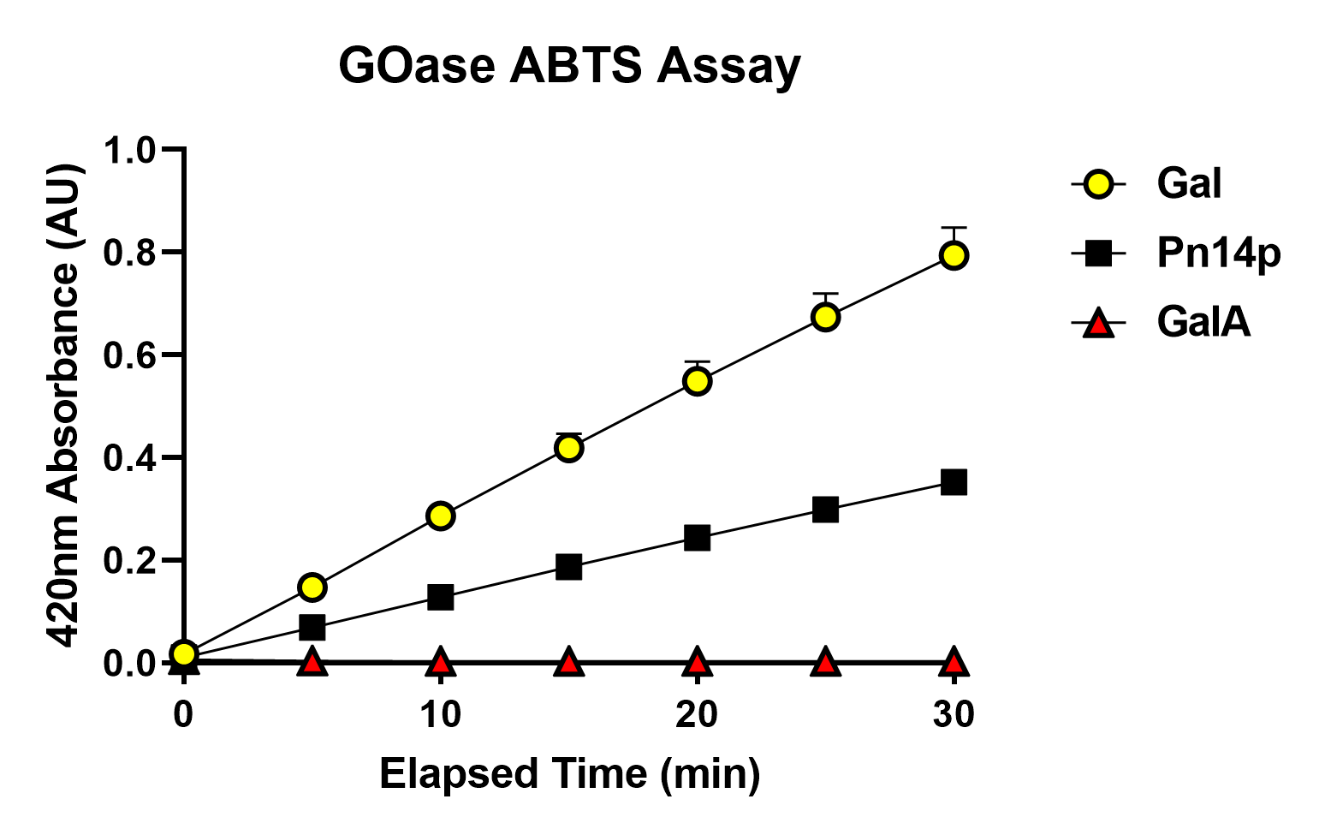


**Supplemental Figure 1.** Galactose Oxidase uses Pn14p as a substrate for oxidation. Reaction time courses were performed in triplicate to determine if GOase would use Pn14p as a substrate for oxidation, with galactose (Gal) and galacturonic acid (GalA) as a positive and negative control, respectively.

**
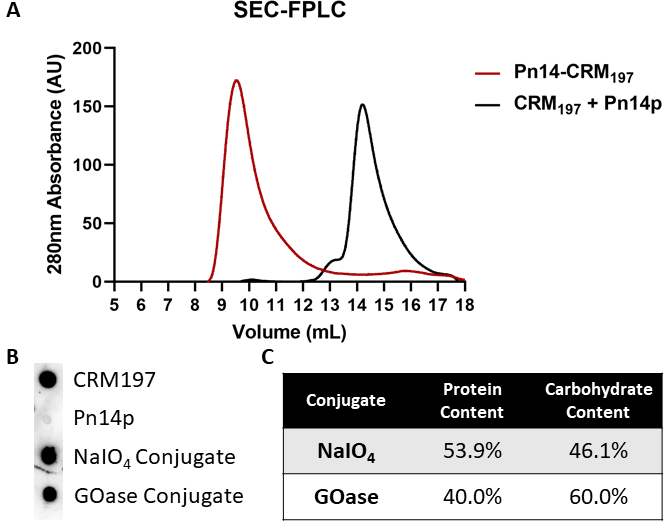
**

**Supplemental Figure 2.** Characterization of glycoconjugate formation. Using both the enzymatic and chemical routes of oxidation, Pn14p-CRM_197_ conjugates were synthesized. A) Purification and validation of conjugation using size-exclusion chromatography compared to a non-conjugated mixture of polysaccharide and protein. B) An anti-CRM dot blotting was performed with mice immunized against the carrier alone to validate the presence of carrier in the conjugate. C) Through BCA and phenol-sulfuric acid assays the protein and carbohydrate contents of both conjugates were determined.
